# Liquid Crystallinity in Epoxy Networks: A Systematic Study of Thermal Conductivity and Structure

**DOI:** 10.3390/polym17192596

**Published:** 2025-09-25

**Authors:** Elias Chalwatzis, Peng Lan, Frank Schönberger

**Affiliations:** 1 Fraunhofer LBF, 64289 Darmstadt, Germany; elias.chalwatzis@lbf.fraunhofer.de; 2 Department of Materials Science and Engineering, University of Illinois, 1304 W Green St., Urbana, IL 61801, USA

**Keywords:** epoxy resin, thermal conductivity, liquid crystalline epoxy, SAXS/WAXS, measurement methods

## Abstract

Epoxy resins are valuable in aerospace, electronics, and high-performance industries; however, their inherently low thermal conductivity (TC) limits applications requiring effective heat dissipation. Recent reports suggest that certain liquid crystalline or partially crystalline epoxy formulations can achieve higher TC, even exceeding 1 W/(m·K). To investigate this, 17 epoxy formulations were prepared, including the commonly used diglycidyl ether of bisphenol A (DGEBA) and two custom-synthesized diepoxides: TME4, which contains rigid aromatic ester linkages with a C4 aliphatic spacer, and LCE-DP, featuring rigid imine bonds. Thermal conductivity was measured using four techniques: laser flash analysis (LFA), modified transient plane source (MTPS), time-domain thermoreflectance (TDTR), and displacement thermo-optic phase spectroscopy (D-TOPS). Additionally, small-angle and wide-angle X-ray scattering (SAXS/WAXS) were performed to detect crystalline or liquid crystalline domains. All formulations exhibited TC values ranging from 0.13 to 0.32 W/(m·K). The TME4–DDS systems, previously reported to be near 1 W/(m·K), consistently measured between 0.26 and 0.30 W/(m·K). Thus, under our synthesis and curing conditions, the elevated TC reported in prior studies was not reproduced, and no strong evidence of crystallinity was observed; indications of local ordering did not translate into higher conductivity. Variations in TC among methods often matched or exceeded the gains attributed to mesophase formation. More broadly, evidence for crystallinity in epoxy thermosets appears weak, consistent with the notion that crosslinking suppresses long-range ordering.

## 1. Introduction

Epoxy resins are widely used in industries ranging from aerospace to electronics due to their excellent mechanical performance, strong adhesion, and superior chemical stability. Their robust nature enables them to function effectively as adhesives, coatings, or encapsulants [1,2,3,4,5,6,7,8,9,10,11]. However, a notable disadvantage is their low thermal conductivity (TC), usually ranging from 0.15 to 0.30 W/(m·K), which can limit their effectiveness in applications requiring efficient heat dissipation [12,13,14,15,16,17,18].

A common route to enhance the thermal conductivity (TC) of epoxies involves adding inorganic fillers, such as silica, alumina, and boron nitride, often at high loadings [19,20,21,22,23,24,25,26]. Highly filled composites can achieve significantly higher thermal conductivity, often exceeding 2 to 3 W/(m·K) [25,27,28,29,30,31,32,33]. However, the required filler content introduces drawbacks, including increased viscosity [34,35], higher density [36,37], increased cost [37], and the risk of filler sedimentation [23], all of which complicate processing, leading to uneven thermal conductivity throughout the material. Thus, there is substantial motivation to discover or develop intrinsically more conductive epoxy formulations that rely less on filler type and content [38,39]. Additionally, increasing the intrinsic TC of the composite matrix can significantly raise the overall TC with identical filler loadings [38,40].

In the broader context of polymeric materials, crystallinity is known to enhance thermal conductivity [41,42,43,44,45,46]. For example, polyethylene can reach values up to 50 W/(m·K) [45,46,47,48,49,50] when polymer chains are highly aligned and form extended crystallites. Notably, recent work by Morikawa et al. [51] reports thermal conductivities exceeding 1 W/(m·K) in spin-coated liquid crystalline polyimide films. However, such values are typically achieved in highly oriented fibers or film structures that do not represent materials processed under typical industrial conditions, such as molding or casting. For thermosets, similar alignment techniques are essentially absent in the literature, as crosslinking severely limits chain mobility. Therefore, the thermal conductivity of thermosets can only be meaningfully compared to that of isotropically processed thermoplastics, where maximized crystallinity yields values up to ~0.5 W/(m·K) in polyethylene [52,53,54,55,56,57]. When polyethylene is crosslinked, molecular mobility is reduced, suppressing crystallization and consequently decreasing thermal conductivity [58,59,60,61,62,63].

Epoxy thermosets undergo crosslinking, which reduces molecular mobility and, in principle, should hinder the formation of large, ordered domains. Nevertheless, some formulations have been reported to exhibit local anisotropy, primarily identified through birefringence in polarized optical microscopy and have been described as exhibiting mesophases—structural states that are intermediate between fully crystalline and fully amorphous [29,30,43,53]. However, birefringence alone cannot distinguish intrinsic ordered phases from other sources of anisotropy, such as residual stress or insufficient mixing, and POM images are difficult to quantify [64]. Some authors refer to these regions as partial crystallinity or liquid crystalline phases, although the constraints imposed by covalent crosslinking would seemingly preclude the formation of extended long-range order known in crystalline polymers [65,66,67,68]. For this study, only difunctional epoxies and difunctional hardeners were combined in a 1:1 stoichiometry to minimize crosslink density and thereby maximize chain mobility. We follow this approach here.

Some publications report not only the presence of liquid crystalline or mesophase structures in their epoxy networks but also semicrystallinity [69,70,71]. A previous report by Lv et al. correlated thermal conductivity to measured crystallinity in epoxy resins [69]. However, a reconsideration of the WAXS data suggests that these results may reflect phase-separated crystallized monomers rather than semicrystalline polymer networks.

While many studies attribute elevated thermal conductivity in liquid crystalline epoxies to increased local ordering, they often lack direct comparisons to a baseline amorphous epoxy measured with the same method [68,72,73,74,75]. Furthermore, side-by-side SAXS/WAXS analyses of reported liquid crystalline formulations against amorphous references are rare. This raises the question of whether the observed ordering is intrinsic to the resin structure and whether it meaningfully enhances thermal transport in a crosslinked thermoset.

TMBP (tetramethyl biphenol epoxy) is regularly cited as forming mesophases responsible for improved thermal conductivity; direct comparisons of the structural analysis to diglycidyl ether of bisphenol A (DGEBA)—a system universally recognized as amorphous—are scarce [65,66,67,68,72,73,76]. An even more striking claim involves the resin TME4 [77,78,79,80,81,82,83,84], for which some publications report thermal conductivity values approaching 1 W/(m·K), identified in one publication as “the highest thermal conductivity measured in an organic isotropic compound.” In these TME4-based systems, the reported high thermal conductivity is attributed to the presence of ordered domains derived from monomers capable of forming liquid crystalline phases. Evidence for ordered domains has been inferred from transmission electron microscopy (TEM) [77] or from peaks in SAXS [78]. In polymer systems, liquid crystalline ordering typically refers to anisotropic domains that retain some orientational order without forming a true crystalline lattice. In crosslinked thermosets, there is no clear definition of what constitutes crystalline or mesophase order, and no standardized approach is reported to quantify such order or relate it to bulk thermal properties. These studies neither quantify the degree of ordering nor compare WAXS/SAXS evidence to known amorphous epoxies. While not all studies can reasonably employ multiple measurement techniques, unusually high TC values in thermosets should ideally be validated against standard references or confirmed with an independent method. The studies cited here do not confirm their high values with a second technique nor benchmark against unequivocally amorphous systems using the same method [77,78,79,80,81,82,83,84].

The epoxy literature contains further discrepancies. A basic formulation, such as DGEBA cured with DDS, has many published thermal conductivity values [74,76,85,86,87,88,89,90,91,92,93,94,95,96,97,98,99,100,101,102,103,104,105,106,107,108,109] ranging from about 0.16 W/(m·K) [89,104] to nearly 0.60 W/(m·K) [92], which already exceeds the incremental gains (typically ~0.2 W/(m·K)) that many liquid crystalline studies claim. This situation raises the fundamental question of what causes such variability. Differences in resin purity, sample thickness, curing schedules, or thermal conductivity measurement methods could potentially produce significant shifts in reported values. These influences have not been systematically dissected, even though it has been repeatedly noted that comparing values obtained from different TC measurement methods can be problematic without standardized protocols [39,110,111,112,113]. Our previous study showed that the TC value varies significantly depending on the measurement method [113]. Nevertheless, data from various sources and methods are often compared at face value [13,53,111,114,115,116], yielding contradictory claims about the potential of these putative liquid crystalline epoxy resins.

In light of these discrepancies, we systematically examined the thermal conductivity of 17 epoxy formulations, ranging from well-established monomers like DGEBA to custom-synthesized di-epoxides such as TME4 and LCE-DP. We tested a variety of curing agents, including both aromatic diamines and flexible diols, with special attention devoted to TME4-based systems, which have been repeatedly cited for achieving conductivities around 1 W/(m·K) [77,78,79,80,81,82,83,84]. To address potential measurement discrepancies, four techniques were employed to evaluate whether the same samples yield consistent results. The goal is to determine whether any epoxy formulation demonstrates the reported high intrinsic conductivity or evidence of partial crystallinity, and to evaluate how variations in testing conditions may contribute to differences in TC values for identical resin-hardener pairs. Additionally, the structure of all resin formulations is investigated with SAXS/WAXS measurements to compare structures described as liquid crystalline side by side with amorphous resin samples. Key conclusions emerging from this work are as follows:

Even for identical formulations, conductivity values can shift significantly depending on the measurement protocol, and only slight increases in TC were observed for more rigid aromatic structures. No clear evidence was found for mesophase formation in the cured epoxies examined when SAXS/WAXS measurements were compared to amorphous epoxies like DGEBA.

In what follows, the experimental design is described in detail, including synthesis, curing procedures, and measurement methods. The results are then presented and discussed in relation to the existing literature, focusing on reconciling contradictory reports and examining the extent to which the different monomers or backbone rigidity can increase the thermal conductivity in epoxy networks.

## 2. Materials and Methods

### 2.1. Materials

Diglycidyl ether of bisphenol A (DGEBA) was obtained as Epilox^®^ A19-02 from Leuna Harze GmbH Am Haupttor 6619, 06237 Leuna, Germany., Diglycidyl ether of 2,2′,6,6′-Tetramethyl-4,4′-biphenol (TMBP) was obtained from Mitsubishi Chemical Europe GmbH Untermainkai 40, 60329 Frankfurt am Main, Germany as jER™ YX4000, LCE-DP and TME4 were synthesized for this work as described below, 1,4-Butanediol (BDO) was purchased from VWR Chemicals Hilpertstraße 20a 64295 Darmstadt, Germany, 1,10-Decanediol (DDO) 1,2,7,8-Diepoxyoctane (DEO), Benzidine (BZD), o-Dianisidine (oDA) and 1 methylimidazole (1 MI) were purchased from Sigma Aldrich Gewerbegebiet Süd, Kappelweg 1, 91625 Schnelldorf, Germany, 4,4′-Diaminodiphenylsulfone (DDS) was purchased from Merck Chemicals Frankfurter Str. 133, 64293 Darmstadt, Germany. All materials were used as received unless otherwise noted.

### 2.2. Synthesis of Epoxy Monomers LCE-DP and TME4

#### 2.2.1. LCE-DP

**Figure 1:**
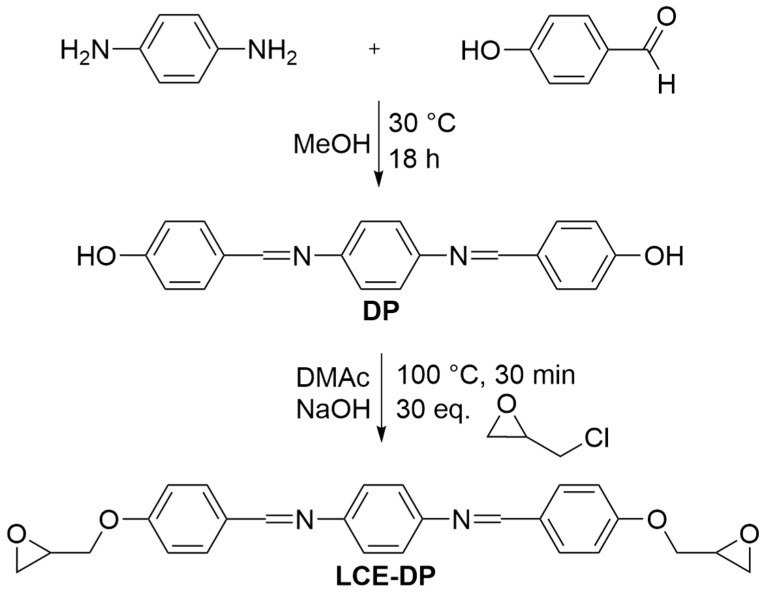
Synthesis of LCE-DP in two steps.

The synthesis of LCE-DP is schematically shown in Figure 1. This method is adapted from [74,75], with modifications introduced in this work to improve yield and product purity.

In a 2 L round-bottom flask equipped with a dropping funnel, nitrogen inlet, and stirring bar, 183.5 g (2 eq, 1.5 mol) of (4-hydroxybenzoic acid) 4HBA and 500 mL of methanol are combined. The flask is then evacuated three times and flooded with nitrogen. A second solution of 81.0 g (1 eq, 0.75 mol) of p-phenylenediamine (pPDA) in 500 mL of methanol is prepared under nitrogen and transferred to the dropping funnel under a nitrogen counterflow. While stirring, the flask is heated to 30 °C. Once the 4HBA is completely dissolved, the pPDA solution is added via the dropping funnel over a period of approximately 30 min. The mixture is stirred for a further 18 h at 30 °C. Due to the large amount of precipitating solid, the solution is not stirred for the entire 18 h. The product is then filtered, and the flask is rinsed twice with 100 mL of methanol. After drying in an air stream for 1 h, the product is washed again with 500 mL of methanol and filtered. The flask is rinsed again twice with 100 mL of methanol, and the product is dried in an air stream for 1 h. The product is then dried in a vacuum oven for one week at 140 °C and 8 mbar. The yield is 225.64 g (95% of the theoretical value).

^1^H NMR (300 MHz, DMSO-d6, δ [ppm]) δ = 10.12 (2H, s, OH), δ = 8.51 (2H, s, CH imine), δ = 7.82–7.77 (4H, tt, aromatic), δ = 7.26 (4 H, s, aromatic), δ = 6.94–6.89 (4H, tt, aromatic).

In a 2 L round-bottom flask equipped with a stir bar and a reflux condenser, 80 g (1 eq.; 253 mmol) of DP and 20.24 g (2 eq.; 506 mmol) of powdered NaOH are combined. Then, 600 mL (30 eq.; 7.6 mol; 702 g) of epichlorohydrin and 800 mL of dimethylacetamide are added. The reaction mixture is stirred and heated to 120 °C, maintaining this temperature for 30 min until complete conversion. To monitor the conversion, a ^1^H-NMR spectrum is recorded. The flask is then cooled to room temperature and placed in a refrigerator overnight at approximately 5 °C to maximize the amount of crystallized product.

The cold solution is filtered, and the collected crystals are washed with 300 mL of methanol. Subsequently, the crystals are washed three more times with 300 mL of water and twice with 300 mL of methanol. The product is dried overnight in a vacuum oven at 40 °C and approximately 20 mbar. The yield is 97.5 g (89% of the theoretical value).

^1^H NMR (300 MHz, DMSO-d_6_, δ [ppm]): δ = 8.60 (2H, s, CH imine), δ = 7.93–7.88 (4H, tt, aromatic), δ = 7.31 (4H, s, aromatic), δ = 7.14–7.09 (4H, tt, aromatic), δ = 4.47–4.42 (2H, dd, CH epoxy), δ = 3.96–3.90 (2H, dd, CH epoxy), δ = 3.41–3.36 (2H, m, CH epoxy), δ = 2.89–2.86 (2H, t, CH_2_ epoxy), δ = 2.76–2.74 (2H, dd, CH_2_ epoxy).

#### 2.2.2. TME4

**Figure 2:**
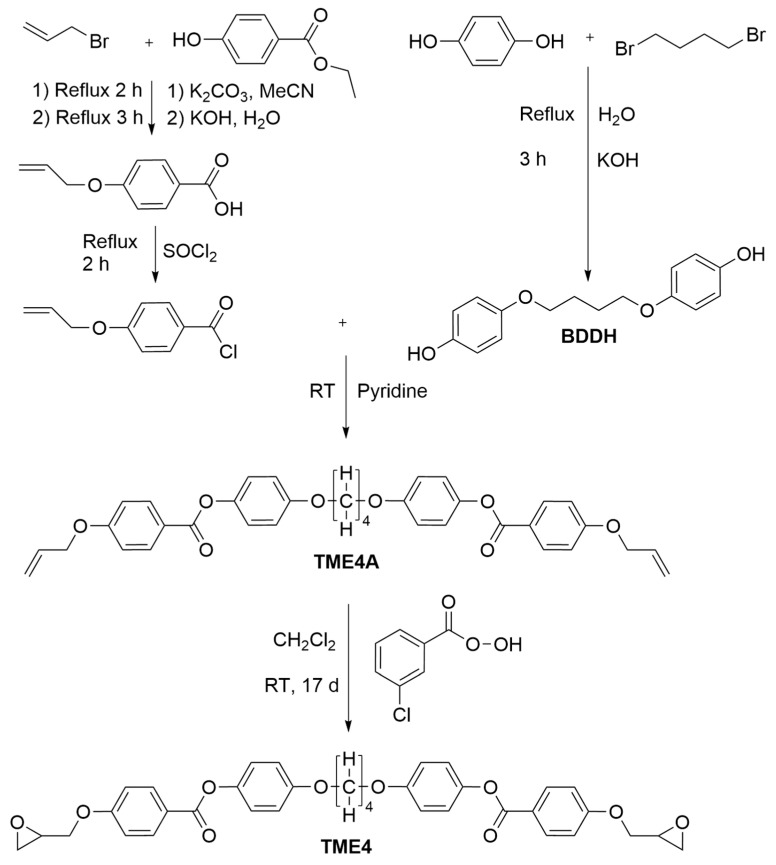
Structural overview of the synthesis of TME4.

The synthesis of TME4, schematically illustrated in Figure 2, combines elements from previously reported procedures [78,117,118] and includes modifications introduced herein to improve yield and product purity.

Synthesis of 4,4′-(1,4-Butanbis(oxy))bisphenol (BDDH)

In a 2 L round-bottom flask equipped with a magnetic stir bar, reflux condenser, dropping funnel, and N_2_ inlet, 440 g (10 eq.; 4 mol) of hydroquinone, 150 mL of distilled water, and 86.4 g (0.4 mol; 1 eq.) of 1,4-dibromobutane are added after the flask has been purged with nitrogen. The reaction mixture is stirred under reflux, and 80 g (3 eq.; 1.2 mol) of 85% KOH dissolved in 80 g of water is added dropwise through the dropping funnel over 15 min. The reaction mixture is initially well stirrable under reflux, but after approximately three-quarters of the KOH solution is added, the reaction mass solidifies and becomes non-stirrable. The reaction mixture is then left under reflux for 3 h before being quenched in 2 L of water while still hot. The aqueous mixture is acidified with 30 g (0.30 mol; 0.75 eq.) of 95% H_2_SO_4_ dissolved in 100 mL of water and stirred overnight at room temperature.

The next day, the suspension is filtered, and the filter residue is washed four times with approximately 750 mL of boiling water to remove remaining hydroquinone. The filter residue is then boiled in 1.2 L of acetone and filtered hot. This process is repeated with 300 mL of boiling acetone. By combining the two fractions (F1 = 64.26 g; F2 = 8.32 g), 72.58 g of crude product is obtained.

For purification, the product is recrystallized from 1750 mL of boiling 99% ethanol. Insoluble residues are removed by hot filtration. The product is washed with 100 mL of 99% ethanol and dried overnight at 80 °C and approximately 20 mbar in a vacuum drying oven. The yield is 65.45 g (60% of theory).

^1^H NMR (300 MHz, DMSO-d_6_): δ = 8.88 (2H, s, OH), δ = 6.78—6.65 (8H, m, aromatic), δ = 3.92–3.88 (4H, t, CH2), δ = 1.82–1.78 (4H, quint., CH2).

Synthesis of p-Allyloxybenzoic Acid

In a 2 L three-necked flask equipped with a magnetic stir bar, N_2_ inlet, and reflux condenser, 83.0 g (0.5 mol; 1 eq.) of ethylparaben, 69 g (0.5 mol; 1 eq.) of K_2_CO_3_, 1500 mL of acetonitrile, and 75 g (0.63 mol; 1.25 eq.) of allyl bromide are added, and the mixture is refluxed for 2 h. Reaction completion is checked by ^1^H-NMR. The reaction mixture is allowed to cool to room temperature, and the remaining inorganic salt is filtered. The salt and flask are rinsed twice with 50 mL of acetonitrile. The acetonitrile fractions are combined, and the acetonitrile is distilled. At the end of the distillation, a vacuum is applied at an oil bath temperature of 130 °C, reaching approximately 5 mbar, to remove most of the acetonitrile.

The residue is combined with 1 L of water and 84.0 g (1.25 mol; 2.5 eq.) of KOH and refluxed with stirring for 3 h. Reaction completeness is verified by ^1^H-NMR. The product is precipitated by adding 165 g of 32% HCl in 1 L of water. After filtration, the product is washed three times with 1 L of distilled water and then recrystallized from 500 mL of ethanol. The product is dried overnight at 80 °C under a vacuum of approximately 20 mbar in a vacuum drying oven.

Yield: 83.51 g (94% of theory).

^1^H-NMR (300 MHz, DMSO-d_6_): δ = 12.61 (1H, s, OH), δ = 7.91–7.87 (2H, tt, aromatic), δ = 7.06–7.01 (2H, tt, aromatic), δ = 6.12–5.99 (1H, m, CH allyl), δ = 5.45–5.37 (1H, qq, CH allyl), δ = 5.31–5.26 (1H, qq, CH allyl), δ = 4.66–4.63 (2H, tt, CH2 allyl).

Synthesis of TME4A

In a 2 L three-neck flask equipped with a magnetic stir bar, reflux condenser, and gas outlet tube, 64 g (0.36 mol; 1.0 eq.) of p-allyloxybenzoic acid is placed and combined with 480 mL (293 g; 2.46 mol; 6.8 eq.) of thionyl chloride. The mixture is heated to reflux, and the resulting gas is directed through an inverted funnel into a solution of 100 g NaHCO_3_ in 2 L of water. The SOCl_2_ is distilled under reduced pressure after no further gas evolution is observed (1–2 h). Initially, the oil bath temperature is set to 40 °C at 100 mbar, and at the end of the distillation, it is raised to 60 °C at 10 mbar.

In a 2 L three-neck flask equipped with a nitrogen inlet and two stoppers, 43 g (0.16 mol; 1 eq.) of BDDH is placed and made inert by three cycles of evacuation and flushing with N_2_. Under a nitrogen stream, 1.1 L of absolute pyridine is added, and the BDDH is dissolved by gentle heating with a heat gun. The solution is added to the previously prepared acid chloride under a nitrogen stream and stirred at room temperature. Subsequently, the pyridine is distilled off at an oil bath temperature of 90 °C and 80 mbar.

The residue is combined with 0.5 L of water, stirred as a suspension, and filtered. The product is washed four times with 0.5 L of water. It is then dried overnight at 80 °C and approximately 20 mbar in a vacuum drying oven.

Yield = 93 g (100% of theoretical yield).

^1^H NMR (300 MHz, CDCl_3_): δ = 8.19–8.13 (4H, tt, aromatic), δ = 7.16–7.11 (4H, tt, aromatic), δ = 7.04–6.93 (8H, tttt, aromatic), δ = 6.16–6.03 (2H, m, CH allyl), δ = 5.51–5.44 (2H, qq, CH allyl), δ = 5.39–5.34 (2H, qq, CH allyl), δ = 4.67–4.64 (4H, qq, CH2 allyl), δ = 4.09–4.06 (4H, t, CH2 butyl), δ = 2.04–2.00 (4H, quint., CH2 butyl).

Synthesis of TME4

First, 75% mCPBA is dried in a crystallization dish in air until most of the added water has evaporated. The resulting mCPBA is estimated to have a concentration of 90% for use in calculations.

In a 1 L amber glass bottle with a magnetic stir bar and screw cap, 10 g (17 mmol; 1 eq.) of TME4A and 6.44 g (34 mmol; 2 eq.) of 90% mCPBA are dissolved in 650 mL of CH_2_Cl_2_. The solution is stirred at room temperature, and after 48 h, 72 h, and 96 h, an additional 3.22 g (17 mmol; 1 eq.) of 90% mCPBA is added. After 12 days, the conversion is complete according to ^1^H NMR.

The DCM is distilled under vacuum at room temperature, and the residue is taken up in 230 mL of ethanol. The suspension is stirred overnight, and the ethanol is filtered. The resulting product is washed three times with 50 mL of ethanol and 30 mL of ethyl acetate. The purity of the product obtained in this manner is sufficient for the preparation of the corresponding resin formulation.

Yield: 7.70 g (73% of the theoretical yield).

^1^H-NMR (300 MHz, CDCl_3_) δ = 8.18–8.15 (4H, d, arom.) δ = 7.14–7.12 (4H, d, arom.) δ = 7.04–6.94 (8H, dd, arom.) δ = 4.38–4.34 (2H, d, CH epoxy) δ = 4.08 (4H, s, butyl) δ = 3.42 (2H, s, CH epoxy) δ = 2.98–2.95 (2H, t, CH epoxy) δ = 2.82 (2H, s, CH epoxy) δ = 2.02 (4H, s., CH2 butyl).

### 2.3. Curing of the Epoxy Formulations

#### 2.3.1. DGEBA and TMBP Cured with BDO, DDO and DDS

In a suitably sized glass vessel with a magnetic stir bar, resin and hardener are weighed. The vessel is covered with aluminum foil and stirred at the required temperature. Once a homogeneous melt or solution has formed, 1-methylimidazole is optionally added as a catalyst.

The prepared resin mixture is then distributed into silicone molds with a diameter of approximately 2.2 cm and a height of approximately 1.5 cm, which have been preheated to the initial curing temperature in a drying oven. Resins cured with DDS are degassed for 5 min at approximately 20 mbar before curing. Silicone molds containing diols in the resin mixture are covered with a Petri dish to minimize the evaporation of the hardener. The curing conditions for DGEBA and TMBP epoxy resins with BDO, DDO and DDS are listed in Table 1.

#### 2.3.2. TMBP + Benzidine/o-Dianisidine

In a PTFE mold with a diameter of 12 mm, a depth of 4 mm, and a bottom thickness of 1 mm or less, 200 mg (0.56 mmol; 1 eq.) of TMBP and 100 mg (0.54 mmol; 1 eq.) of benzidine, or alternatively 181 mg (0.51 mmol; 1 eq.) of TMBP and 119 mg (0.49 mmol; 1 eq.) of o-dianisidine, are weighed. The powders are thoroughly mixed and placed in a vacuum drying oven at 180 °C.

After the mixture melts within 2 min, a vacuum of approximately 20 mbar is applied for 4 min to degas the test specimen. The drying oven is then vented, and the test specimen is cured at 180 °C for 3 h.

To facilitate removal of the test specimen from the mold, it is first cooled in liquid nitrogen and then brought back to room temperature by warming it with water.

#### 2.3.3. LCE-DP + BDO

In a mortar bowl, 4.29 g (10 mmol; 1 equiv) of LCE-DP and 0.92 g (10 mmol; 1 equiv.) of 1,4-butanediol are weighed and ground into a homogeneous mass. The mixture is poured into a silicone mold with a diameter of approximately 2.2 cm and a height of approximately 1.5 cm.

The mold is placed in a vacuum drying oven preheated to 200 °C for 20 min. During this time, the resin mixture melts, and the pressure is immediately reduced to approximately 200 mbar to degas the mixture. After a total of 22 min in the oven, the drying oven is vented to restore atmospheric pressure. The sample is then cured for 4 h at 200 °C.

#### 2.3.4. LCE-DP + DDO

In a mortar bowl, 3.68 g (9 mmol; 1 equiv) LCE-DP and 1.50 g (9 mmol; 1 equiv) of 1,10-decanediol are weighed and ground into a homogeneous mixture. The resulting mixture is poured into a silicone mold with a diameter of approximately 2.2 cm and a height of approximately 1.5 cm.

The mold is then placed in a vacuum drying oven preheated to 200 °C for 23.5 min. During this time, the sample melts, and the pressure is immediately reduced to approximately 25 mbar. After a total of 24.5 min in the oven, the drying oven is vented, and the sample is cured for 2 h at 200 °C.

#### 2.3.5. LCE-DP + DDS

A thoroughly mixed and ground mixture of 284 mg (0.7 mmol; 1 equiv) LCE-DP and 166 mg (0.7 mmol; 1 equiv) DDS is placed into a PTFE mold with a diameter of 12 mm, a depth of 4 mm, and a bottom thickness of 1 mm or less. The mold is placed in a vacuum drying oven preheated to 200 °C.

After the mixture melts within 8–10 min, a vacuum is applied for 60 to 90 s to degas the sample. Before the sample cures, the vacuum drying oven is vented, and the sample is cured for 2 h at 200 °C.

To simplify the removal of the sample from the mold, it is cooled in liquid nitrogen and then warmed back to room temperature using water.

#### 2.3.6. TME4 + DDS for Modified Transient Plane Source (MTPS)

Different processes were used to make the TME4 + DDS sample for two reasons: (1) to assess how the preparation of the sample affects the thermal conductivity and (2) because larger samples needed for MTPS measurements could not be produced in the same manner as the smaller samples, since the gel time was too short to remove all the air bubbles before it cured. Therefore, the following process was used to prepare a larger sample for the MTPS measurement: In a mortar bowl, 1.15 g of TME4 (2 mmol; 1 eq.) and 0.45 g (2 mmol; 1 eq.) of DDS are weighed and ground into a homogeneous mass. The mixture is placed in a pressing tool with a diameter of 20 mm and pressed at 100 bar. The pressure is then reduced to 20 bar, and the tool is heated to 115 °C. As the tool heats, the pressure increases to 22.5 bar.

Subsequently, the temperature is raised by 1 °C every two minutes until it reaches 140 °C. During this process, the pressure decreases to 21 bar. The temperature is held constant at 140 °C for 30 min, after which it is again increased by 1 °C every two minutes until 150 °C is reached. During this phase, the pressure further decreases to 19 bar.

Next, the temperature is raised to 180 °C over 20 min, while the pressure decreases to 17.5 bar. The temperature is maintained at 180 °C for 1 h, during which the pressure drops further to 14 bar.

The sample, due to its flat surface, can be used directly for thermal conductivity measurements using the MTPS method.

#### 2.3.7. TME4 + DDS for LFA, TDTR and D-TOPS

In a PTFE mold with a diameter of 12 mm, a depth of 4 mm, and a bottom thickness of 1 mm or less, 216 mg (0.3 mmol; 1 equiv) of TME4 is weighed. On a second Teflon plate, 87 mg (0.4 mmol; 1 equiv) of DDS is weighed, including a 3 mg excess to compensate for transfer losses. The TME4 and DDS are melted at 180 °C in a vacuum drying oven under atmospheric pressure.

The two molten monomers are degassed under a vacuum of approximately 20 mbar. After careful venting, the molten DDS is transferred into the TME4 melt. Gently tapping the Teflon plate on the PTFE mold ensures that the DDS drop falls directly into the TME4 melt. Using a PTFE rod preheated to 180 °C and held with tweezers, the mixture is thoroughly stirred for approximately 2 min.

A vacuum of up to 20 mbar is then reapplied for an additional 2 min to finalize the degassing of the resin mixture before the test specimen cures. The prepared specimen is cured for an additional 18 h at 180 °C.

To facilitate removal of the specimen from the mold, it is first cooled in liquid nitrogen and then brought back to room temperature using water.

### 2.4. Thermal Conductivity Measurement Methods

#### 2.4.1. Modified Transient Plane Source (MTPS)

For the measurement of TC, the TRIDENT device from C-Therm Technologies Ltd. 40 Crowther Ln, Fredericton, NB E3C 0J1, Canada was utilized. The measurements were performed using the modified transient plane source (MTPS) sensor. During the measurement, the temperature at the sensor increased by 1–2 °C, and the temperature difference over time was recorded. The test samples required a smooth and flat surface to generate reproducible results. To achieve this, the surface of the samples was ground using the Abramin grinding and polishing device from Struers Carl-Friedrich-Benz Straße 5, 47877 Willich, Germany before each measurement. The grinding paper used had a grain size of 16 μm. Water was used as the contact medium between the sensor and the sample. While water has a significantly higher thermal effusivity than the epoxy samples, it was used to ensure consistent thermal contact. Deviations from surface flatness were minimized as much as possible, though they may still contribute to uncertainty in the measurement. The test samples were weighted down with a 500 g weight to improve the contact between the sensor and each sample. For each resin formulation, three samples were made in the described manner, and each sample was measured three times on each side. Since this required large samples compared to the other measurement methods, and not all the resins could be processed into such a shape, or there was not enough starting material available, not all samples could be measured using this method.

The measurement raw data were the temperature at each time during the measurement. These data were processed by plotting ΔT against the square root of the time. The inverse slope of this curve between 0.7 and 1 in units of √t is then entered into a calibration curve to get the thermal effusivity. Pyrex glass (effusivity: 1440 $\frac{W\sqrt{s}}{m^{2}K}$) and Pyroceram (effusivity: 2950 $\frac{W\sqrt{s}}{m^{2}K}$) are used as the calibration standards. Although these reference materials have higher effusivity than the epoxy samples, they were chosen for consistency with manufacturer calibration procedures. The calibration curve is obtained by plotting inverse slope versus effusivity for the two standards. By squaring the thermal effusivity and dividing by the volumetric heat capacity, the thermal conductivity is derived. The volumetric heat capacity is derived by multiplying the specific heat capacity with the density. The measurements of these last two values are described in the corresponding sections of the experimental part. The average of all these measurements was calculated, which improved the measurement precision and helped reduce random measurement variability, with overall variation across samples typically within ±0.01 W/(m·K). C-Therm quotes an accuracy better than 5% for the device.

#### 2.4.2. Laser Flash Analysis

For laser flash TC measurements, the LFA 427 device from NETZSCH-Gerätebau GmbH, Wittelsbacherstraße 42, 95100 Selb, Germany was employed. Sample cylinders with a diameter of 12.6 mm and a height of 1 mm were milled from the test specimen prepared for the MTPS method. In general, samples that could not be measured using the MTPS method could also not be measured with the LFA method. However, there are five exceptions where the samples could be prepared using a smaller PTFE mold (as discussed in the previous sections of this chapter). For other samples, material availability or processing challenges prevented these measurements. The sample thickness was measured using a micrometer at five different locations per specimen, and the average value was used for thermal diffusivity calculations. The sample diameter was measured in the same manner using a digital caliper. The primary sources of uncertainty in the laser flash measurement are estimated to be the thickness measurement and the volumetric heat capacity, which was calculated from separately measured density and specific heat capacity values.

The test specimens were coated on both sides with graphite varnish (Graphit 33 from CRC-KONTAKTCHEMIE Südring 9, 76473 Iffezheim, Germany) to ensure complete absorption of the laser pulse. The coating thickness was not measured; however, care was taken to apply a uniform and sufficiently opaque layer on each specimen. During the measurement, the sample was irradiated with a light pulse on one side, and the temperature increase over time on the other side was determined. The faster the increase in temperature on the other side of the sample, the higher the thermal diffusivity. The corresponding software calculated the thermal diffusivity, from which the TC was derived by multiplying by the density and specific heat of the sample. The measurements of these last two values are described in the corresponding sections of this experimental section. Three samples were made for each resin formulation, and they were measured three times, flipping them upside down after each measurement. The average of all these values was calculated, improving measurement precision and helping to reduce random measurement variability, with overall variation across samples typically within ±0.01 W/(m·K). NETZSCH, Wittelsbacherstraße 42, 95100 Selb, Germany quotes an accuracy of ±3% for the thermal diffusivity. The polyimide Vespel^®^ was used as a standard reference material.

#### 2.4.3. Time-Domain Thermoreflectance (TDTR)

Epoxy samples are prepared through mechanical polishing to achieve a surface with a high degree of optical reflectivity, specifically ensuring that the diffuse reflectivity is less than 3% of the specular reflectivity. The polishing process begins with sanding the samples using sandpaper of sequentially smaller grain sizes. Starting with 1000 grit sandpaper, the grit size is progressively refined using 2000 grit, 3000 grit, and finally 5000 grit. To achieve a final polish, ROTWEISS, Sandgraben 8, 88142 Wasserburg (Bodensee), Germany Top-Glanz Anti-Hologramm-Politur, which has a grit equivalent of 25,000, is applied. Subsequently, an Al transducer film of ~80 nm is sputtered onto the surface under high vacuum (<10^−7^ torr) conditions. The samples are utilized for thermal conductivity measurements using time-domain thermoreflectance (TDTR) and displacement thermo-optic phase spectroscopy (D-TOPS). The experimental temperature is maintained at room temperature, ranging from 22 to 23 °C.

The TDTR technique measures thermal effusivity (*k*^1/2^*C*^1/2^) and is particularly sensitive to the through-plane thermal conductivity of the studied samples. The experimental setup follows previously reported configurations, with the pump/probe laser set to 785 nm and configured to output 2 mV and 1 mV for the probe to minimize local steady-state heating (which is approximately 15 K in this case). The laser modulation frequency is set to 9.3 MHz, and the beams are focused and overlapped using a 10x objective lens with a 1/*e*^2^ radius of 4.9 μm. The signals are recorded as a function of time delay, linearly from −20 ps to 80 ps for 100 datapoints (with 1 point per second), followed by scans from 80 ps to 3600 ps for 64 datapoints. Fitting is generally performed from 200 ps to 3600 ps using established thermal models reported in the literature [119,120]. The Al thermal conductivity is determined through the deposition of aluminum on a fused silica substrate, along with measurements of electrical conductivity using a four-point probe method.

#### 2.4.4. Displacement Thermo-Optic Phase Spectroscopy (D-TOPS)

D-TOPS measures thermal diffusivity (*k/C*) and is sensitive to the in-plane thermal conductivity of the studied samples. The experimental setup follows previously reported configurations, with the pump/probe laser set to 780 nm and 670 nm, respectively, configured to output 0.6 mV for the pump and 0.8 mV for the probe, using a 5 × objective lens with a 1/*e*^2^ radius of 11.2 μm, resulting in a local steady-state heating of approximately 5 K. The beams are offset by 12.6 μm. Signals are detected as a function of frequency, ranging from 10,000 Hz to 100 Hz, yielding a total of 40 data points (with 1 point per second). Fitting is performed using established thermal models reported in the literature [121]. The beam spot size and offset are verified by standard CaF_2_ sputtered with an Al layer of 74 nm prior to measuring the sample.

Since no external force field is applied and the samples are used for thermal conductivity measurements in their synthesized state, we assume that the thermal conductivity of the studied epoxies is isotropic. Therefore, by combining TDTR and D-TOPS, both thermal conductivity and volumetric heat capacity can be obtained with an uncertainty of 5%. When integrated with TDTR data, the D-TOPS approach enables the extraction of both thermal conductivity and volumetric heat capacity in a self-consistent manner. However, the volume probed by each method differs; thus, if the thermal conductivity is not spatially homogeneous, this approach may fail. TDTR is sensitive only to the top 100 nm of the sample, while the probing depth of D-TOPS is on the order of the spot size, approximately 10 μm in this case. In contrast, LFA measures the average property through the thickness of the 1 mm sample, and the MTPS approach measures the properties within the thermal penetration depth during the measurement time, which is approximately 300 μm for the materials studied here. Although this methodology avoids separate DSC or density measurements and may reduce associated uncertainties, we do not attempt to rank the accuracy of this method against other approaches, such as the combination of C-Therm and laser flash analysis (MTPS and LFA). Instead, our focus is on documenting how different methods, even when applied carefully, yield varying values for the same material.

#### 2.4.5. Heat Capacity

The thermal transition behavior of the epoxies is measured using Differential Scanning Calorimetry (DSC, with a TA 2500 instrument) over a temperature range from −20 °C to 200 °C at a heating/cooling rate of 10 °C/min. Hermetic pans and lids are utilized to load the samples with each measurement having a mass of approximately 7 mg. The specific heat (*C_sp_*) is also obtained from DSC, calibrated using a standard sapphire sample and verified with a 30 kDa PMMA (PDI = 1.02) prior to the measurements, resulting in an error margin of 5%. By combining the specific heat capacity with the sample density, the volumetric heat capacity of the epoxies can be calculated.

#### 2.4.6. Density

For the density measurements, the samples were first weighed, and afterwards their volume was determined by submerging the samples in water with a drop of soap, while they were held up by a copper wire from above. The mass value displayed on the scale in grams (g) can be directly converted to the sample volume in milliliters (mL) at a 1:1 ratio, assuming the density of water is 1 at room temperature. The volume of the copper wire was previously determined in the same way and was subtracted from the sample volume. Dividing the determined volume by the mass results in the mass density.

### 2.5. SAXS/WAXS Measurements

The morphology of the studied epoxies is characterized using a custom-built transmission small-angle X-ray scattering (SAXS) and wide-angle X-ray scattering (WAXS) setup at the University of Illinois. A Xenocs GeniX3D Cu Kα X-ray source (1.54 Å) and a Pilatus 2D detector are employed. The power supply is set to 50 kV and 0.6 mA. The baseline beam path is maintained under vacuum for the SAXS detector and in air for the WAXS detector. The samples used for these measurements are the same as those used for the TDTR and D-TOPS experiments, sanded down to a thickness of 0.5 mm. The sample-to-detector distance and absolute signal intensity are calibrated prior to the measurements, and corresponding sample X-ray spectra are collected with a 30-min exposure time at room temperature. The FIT2D V12.012 software is utilized to process the 2D scattering patterns, which have a size of 75 μm × 75 μm, allowing for a radially-averaged integration of intensity versus scattered wave vector to be obtained and plotted.

## 3. Results

### 3.1. Overview of the Results

Seventeen epoxy formulations were selected to assess how molecular structure and measurement protocols affect thermal conductivity (TC) values, particularly in the context of reported liquid crystalline or semi-crystalline phases. These formulations were created by combining epoxy monomers with varied chemical structures and curing agents of different rigidity, resulting in a broad range of molecular architectures.

Initially, the structures of the monomers are presented along with the rationale for selecting each resin and hardener for this investigation. A comparison of the measured thermal diffusivities, effusivities, and resultant thermal conductivities illustrates potential discrepancies between different measurement methods. Attention is then directed toward verifying whether TME4-based formulations can genuinely reproduce the high conductivity claims reported in the literature. To explore whether partial crystallinity or liquid crystalline phases might account for any observed differences, SAXS/WAXS comparisons of the cured networks are discussed.

This approach facilitates a direct comparison among resin formulations while examining how both monomer structure and measurement protocol influence the reported values. We investigate whether any formulation achieves the high thermal conductivities previously attributed to ordered phases in epoxy networks and how methodological factors may help explain the broad variation observed in the literature. Rather than attempting to refute prior claims, this work emphasizes reproducibility across measurement methods and seeks to establish whether the previously reported values can be independently reproduced.

### 3.2. Investigated Resins and Curing Agents

**Figure 3:**
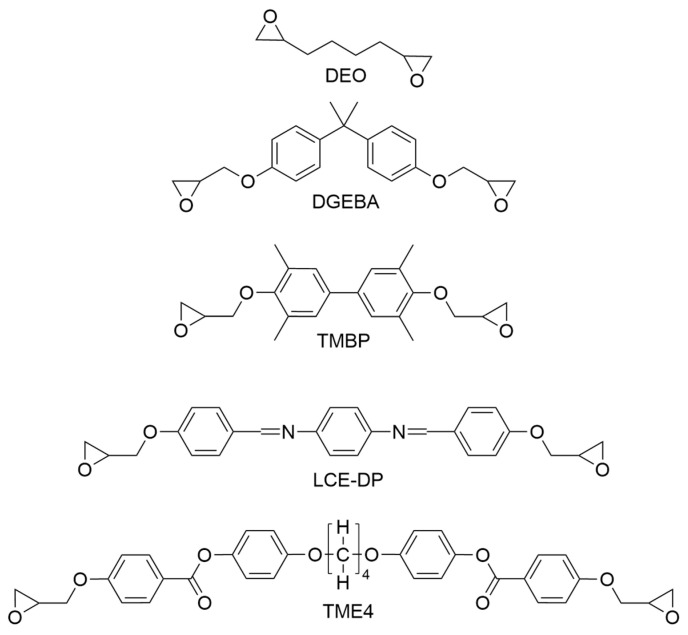
Epoxy monomers investigated in this study: DEO (1,2,7,8-diepoxyoctane), DGEBA (diglycidyl ether of bisphenol A), TMBP (diglycidyl ether of 2,2′,6,6′-tetramethyl-4,4′-biphenol), LCE-DP (N,N′-(1,4-phenylene)bis(1-(4-(oxiran-2-ylmethoxy)phenyl)methanimine)), TME4 (butane-1,4-diylbis(oxy))bis(4,1-phenylene) bis(4-(oxiran-2-ylmethoxy)benzoate)).

The epoxy monomers are displayed in Figure 3:

DEO is an aliphatic epoxy that provides a flexible, low-viscosity option, included to facilitate a comparison between aliphatic and aromatic backbones.

DGEBA is a widely used industrial epoxy and serves as a baseline for comparison against the less conventional and custom-synthesized epoxy monomers.

TMBP was selected due to its industrial availability and its marketing as a crystalline epoxy. Several publications indicate that TMBP can enhance TC by forming ordered structures [65,66,67,68,72,73,76].

LCE-DP features a rigid backbone structure that some literature has associated with liquid crystallinity and enhanced thermal conductivity. Its synthesis requires only two steps, making it more accessible than TME4 [25,29,30,74,75,122,123].

TME4 has been reported to achieve thermal conductivity values nearing 1 W/(m·K) when cured with DDS or DDM. Although it is more challenging to synthesize (requiring five steps), the high conductivity reported in previous studies underscores its significance for this investigation [77,79,80,81,82,83,84].

**Figure 4:**
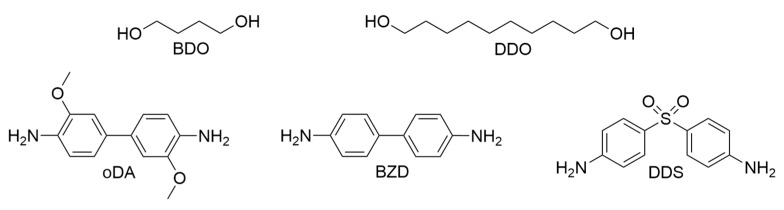
Curing agents used in this study: BDO (1,4-butanediol), DDO (1,10-decanediol), oDa (3,3′-dimethoxybenzidine), BZD (benzidine), DDS (4,4′-diaminodiphenylsulfone).

The curing agents used are shown in Figure 4:

DDS (4,4′-diaminodiphenylsulfon) is frequently employed in high-performance applications and is widely referenced in the literature on thermally conductive epoxies, enabling direct comparisons to existing studies [29,30,69,74,75,76,114,115,116].

BDO (1,4-butanediol) and DDO (1,10-decanediol) are flexible aliphatic diols. They have proven useful when formulating with LCE-DP, which melts at 194 °C and self-catalyzes the curing reaction with the azomethine nitrogen group of LCE-DP. This high melting point left less than one minute of workable liquid phase when using aromatic amines (e.g., DDS), making sample preparation challenging. In contrast, BDO and DDO facilitated easier sample fabrication due to their lower reactivity. However, BDO and DDO are volatile, requiring 1-methylimidazole as a catalyst to ensure proper curing with DGEBA and TMBP (not with LCE-DP) at lower temperatures before they evaporate. Because of their volatility, BDO and DDO were partially lost during high-temperature curing. This was verified by weighing the samples before and after curing, which indicated deviations from the intended stoichiometry. However, additional test samples were prepared with intentionally varied stoichiometries, and these showed no significant change in thermal conductivity. This suggests that the precise curing ratio had minimal influence on the final TC values under the tested conditions. DEO combined with BDO or DDO did not yield solid samples, so those pairs were excluded from thermal testing. Similarly, DGEBA and TMBP cured with DDO exhibited glass transition temperatures near room temperature, prohibiting certain measurements (TDTR, D-TOPS).

BZD (benzidine) and oDa (3,3′-dimethoxybenzidine) were included as more rigid aromatic amines. This allowed for the comparison of flexible versus rigid curing agents to investigate how the overall network stiffness impacts thermal behavior.

Not all hardeners were used to cure all resins due to technical limitations (processibility, reactivity, volatility) and to prioritize combinations that allow meaningful comparison with the literature.

### 3.3. Comparison of the Measurement Methods

Four techniques were employed to characterize thermal conductivity, along with supporting measurements of heat capacity, density, and glass transition temperature:

- Laser flash analysis (LFA)
- Modified transient plane source (MTPS)
- Time-domain thermoreflectance (TDTR)
- Displacement thermo-optic phase spectroscopy (D-TOPS)

Table 2 lists all the thermal diffusivity and thermal effusivity values obtained from the corresponding measurement methods and samples that were measurable with each technique. Additionally, the specific heat capacity and density values necessary for calculating thermal conductivities are also included.

A direct comparison of the effusivities measured by TDTR and MTPS reveals systematic discrepancies, with TDTR consistently yielding lower values. For the TMBP + DDS combination, the MTPS value exceeds the TDTR result by a factor of ~1.4, corresponding to a 1.6-fold difference in thermal conductivity. This discrepancy falls well outside the nominal error bounds of either technique, indicating that it cannot be attributed solely to measurement uncertainty. A possible explanation is spatial inhomogeneity: TDTR probes only the top ~100 nm of the sample, while MTPS samples within a thermal penetration depth of several hundred microns. Thus, differences between the near-surface region and the bulk could account for the observed divergence. A similar trend is noted in the diffusivity measurements, where LFA consistently reports larger values than D-TOPS. The maximum discrepancy approaches a factor of 1.5 for TMBP + BZD, again exceeding the combined error bars of the two methods. Unlike the effusivity case, no obvious explanation for this difference is apparent. These comparisons highlight the significant method-dependent variability and emphasize the need for caution when comparing thermal conductivity data obtained using different experimental techniques.

**Figure 5:**
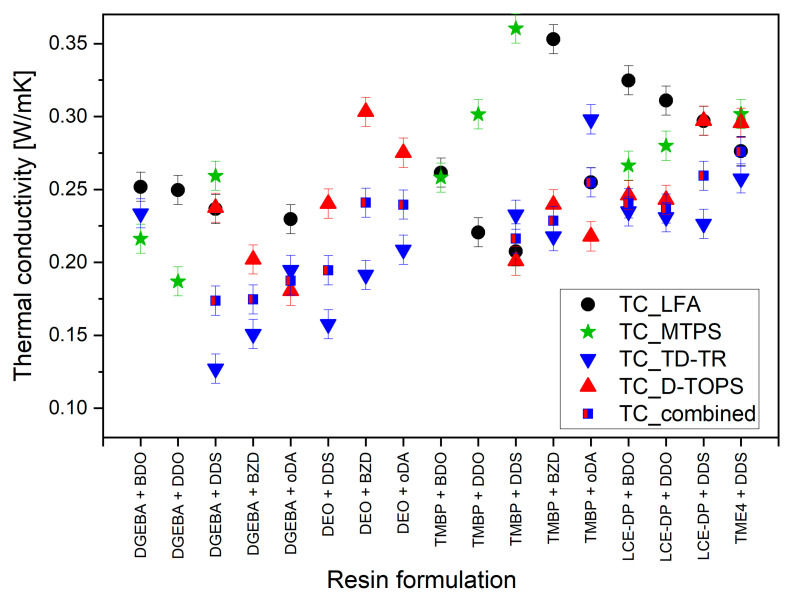
Thermal conductivities of all resin formulations measured with the four different measurement methods, and the TC calculated using the combination of D-TOPS and TDTR (TC_combined).

The thermal conductivities calculated from the values of all the samples in Table 2 across the measurement methods are plotted in Figure 5.

Most formulations exhibited thermal conductivities in the range of 0.2–0.3 W/(m·K), but notable variations emerged depending on the measurement method. Below, we discuss these differences along with the strengths and limitations of each technique.

The MTPS method and LFA tended to yield slightly higher TC values, while TDTR consistently produced the lowest TC values for these samples. In contrast, D-TOPS yielded results that fell between the values obtained from MTPS/LFA and TDTR. The combined values calculated from the TDTR and D-TOPS measurements without using externally measured heat capacity and density were between the values obtained using externally measured densities and heat capacities (Table 2), as expected.

Coating with graphite spray in LFA can be more challenging for aliphatic-rich samples due to potential swelling from the solvent, which complicates the application of a homogenous, opaque layer of graphite. In contrast, TDTR and D-TOPS require aluminum sputtering on polished surfaces, representing a different type of sample preparation requirement. Flexible samples (those with longer aliphatic chains and low T_g_) were more difficult to shape into precise geometries and polish, which could further affect measurements, especially for LFA and MTPS.

Despite the known differences between methods, repeated measurements using the same technique yielded consistent results across separately prepared samples, even when variations in curing conditions or stoichiometry were present. Any significant outliers in thermal conductivity values were investigated, but ultimately no formulation exhibited TC values approaching 1 W/(m·K). While our measured density for TME4-based samples (1.3 g/mL) was somewhat lower than previously reported values (~1.4 g/mL) [78]; this difference alone is unlikely to account for the significant thermal conductivity gap. A more detailed analysis of this discrepancy follows in the next section.

While thermal conductivity is a well-defined material property, numerous studies have reported method-dependent disparities in measured values, reflecting the influence of technique-specific assumptions and sample conditions [39,110,111,112].

### 3.4. Comparison of the Heat Capacities

The heat capacities are determined using three different methods:

(1) The LFA reference comparison;
(2) DSC;
(3) Dividing the thermal effusivity of TDTR by the square root of the thermal diffusivity measured by D-TOPS.

**Figure 6:**
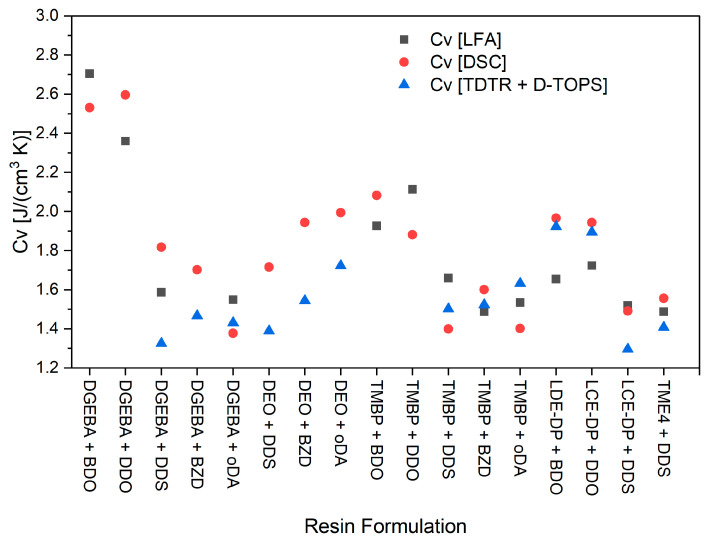
The volumetric heat capacities for all the different resin samples measured using the different measurement methods, LFA reference, DSC, and TDTR + D-TOPS combination.

Using the last method allows for the direct calculation of thermal conductivity without relying on externally measured density and heat capacity, assuming that thermal conductivity is isotropic and spatially homogenous. Therefore, it is particularly interesting to compare these calculated values directly with the conventionally measured values obtained using DSC. However, a direct comparison is not feasible because DSC measures specific heat capacity, which results in volumetric heat capacity when combined with density. Consequently, the DSC values in Table 2 are multiplied by the density values in Table 2 to obtain the volumetric heat capacities for accurate comparison. The same applies to the specific heat values derived from LFA. All the values are displayed in Figure 6:

It is evident that the measurement using DSC yields the highest values of volumetric heat capacity, while the combined method of TDTR and D-TOPS tends to produce the lowest values. This trend was observed even for most high-T_g_ systems, indicating that method-dependent factors beyond T_g_ also contribute. The values measured using the reference method in LFA appear to fall somewhere in between. A similar discrepancy exists between the heat capacity measurements and the thermal conductivity values for all samples, which is certainly one significant source of error. While part of this difference may arise from formulations with low T_g_, additional method-dependent effects seem to contribute and are beyond the scope of this work. The unusually high heat capacity values for DGEBA/BDO and DGEBA/DDO likely result from their glass transition temperatures being below room temperature, leading to increased configurational contributions.

### 3.5. Thermal Conductivity in TME4/DDS

Among the formulations tested, TME4 epoxy cured with DDS or DDM has been highlighted in the literature as potentially having a thermal conductivity around 0.8–1.0 W/(m·K) [77,78,79,80,81,82,83,84]. In the present study, a TME4/DDS formulation was prepared and characterized multiple times using all four measurement methods. All results for TME4/DDS fell within a relatively narrow range of 0.26–0.30 W/(m·K) across all four measurement techniques and multiple separately prepared samples. This consistency reflects both the relatively low intrinsic thermal conductivity of the material and the reproducibility of the individual measurement methods.

Reports of ~1 W/(m·K) for TME4 cured with DDS (or DDM) originate from only two research groups, primarily authored or co-authored by Yoshitaka Takezawa [77,79,80,81,82,83,84]. These studies largely rely on a single set of measurements, and independent replications outside these groups have not been reported. While thermal conductivity is, in principle, independent of the measurement technique, practical differences in sample preparation and method assumptions can lead to variations. This underscores the importance of confirming unusually high reported values using multiple approaches, as was done in this study. In a more recent publication led by Prof. Cahill [78], which also reported high thermal conductivity for TME4/DDS, the authors employed TDTR and I-TOPS measurements. However, in our current work on TME4/DDS using TDTR and D-TOPS, we did not reproduce the previously reported high conductivity in newly synthesized TME4 samples, despite conducting the experiments under identical conditions in the same laboratory using the same equipment. In our investigation, we implemented the following measures to attempt to replicate the high conductivity values reported in prior studies:

- Purity of the TME4 monomer was checked via ^1^H NMR and compared to earlier batches from the previous study [78]. No additional peaks or changes in peak integrals larger than experimental noise were observed.
- Recrystallization solvents for TME4 (e.g., toluene, dimethylacetamide) were varied, with no significant impact on the final measured thermal conductivity.
- Purity of DDS: Verified by melting point analysis in DSC and by NMR; no deviations from standard reference data.
- Curing protocol variations:
  ∘ Hot press vs. oven curing.
  ∘ Monomers melted individually before mixing vs. mixing powders directly at room temperature.
  ∘ Curing temperature (e.g., 160–200 °C) and curing time (e.g., 2–20 h) varied.
- However, in DSC measurements, the melting endotherm overlaps the curing exotherm across the tested curing-temperature range, limiting independent adjustment of the curing temperature.

Despite these changes, the measured conductivity consistently remained between 0.26 and 0.30 W/(m·K). Densities, heat capacities, and T_g_ measurements fell within the normal ranges for an aromatic epoxy–amine system and were comparable across all samples regardless of the preparation method.

The primary difference between the previously reported values and those measured in this study lies in the reported thermal diffusivity. Sample thickness was not controlled in the earlier measurements, which may influence the analysis of thermal transport. However, even taking this into account, we were unable to reproduce the previously reported high values under any of the controlled conditions employed in this work.

Based on these evaluations, we concluded that the previously reported ~1 W/(m·K) values could not be reproduced under any of the conditions used in this study. No combination of TME4 monomer purity, curing conditions, or measurement refinements yielded conductivity values significantly above ~0.3 W/(m·K) in our experiments.

### 3.6. Structural Analysis via SAXS/WAXS

**Figure 7:**
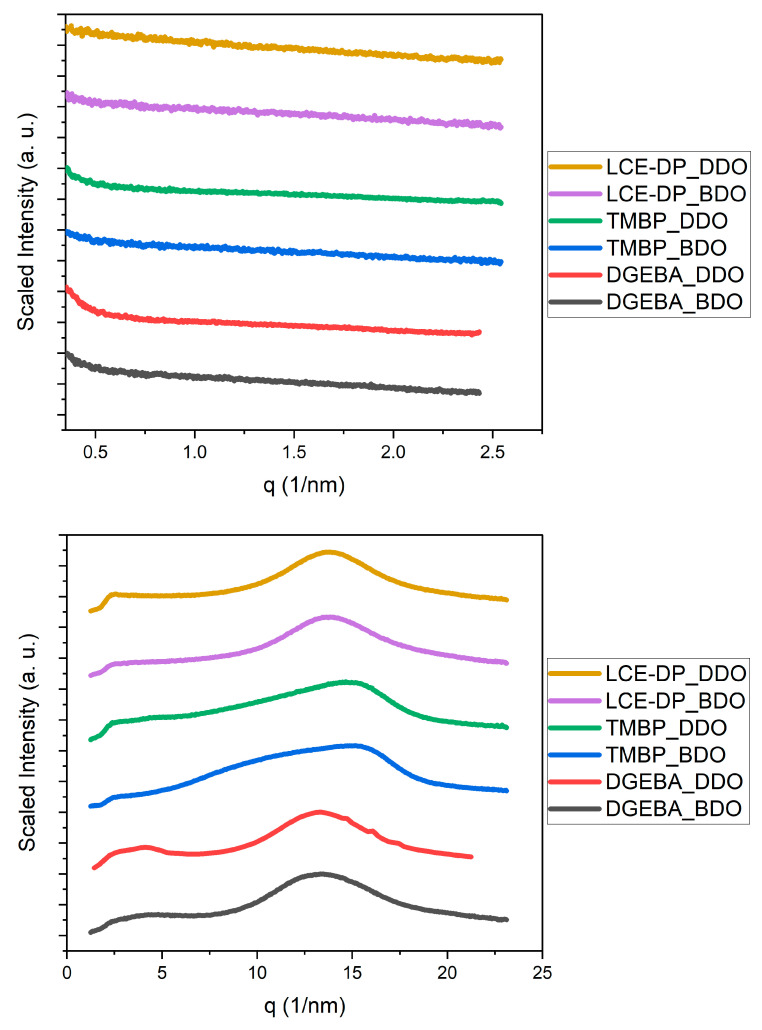
SAXS (**top**) and WAXS (**bottom**) patterns of LCE-DP, TMBP, and DGEBA cured with DDO and BDO.

**Figure 8:**
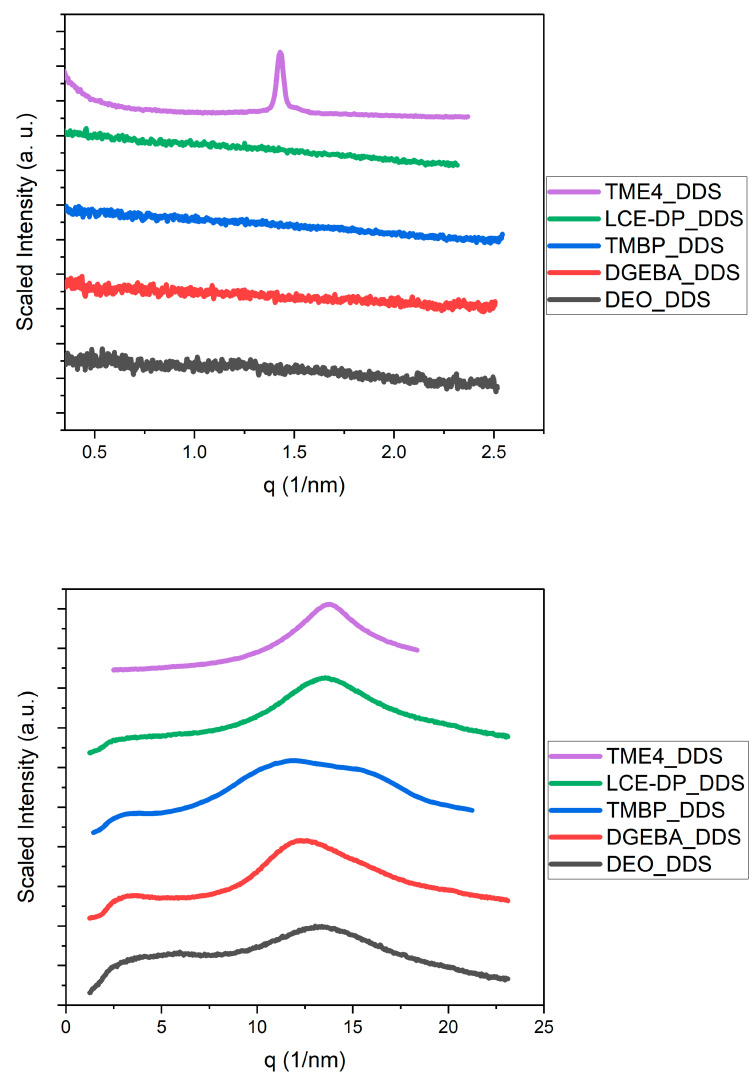
SAXS (**top**) and WAXS (**bottom**) patterns of TME4, LCE-DP, TMBP, DGEBA, and DEO cured with DDS.

**Figure 9:**
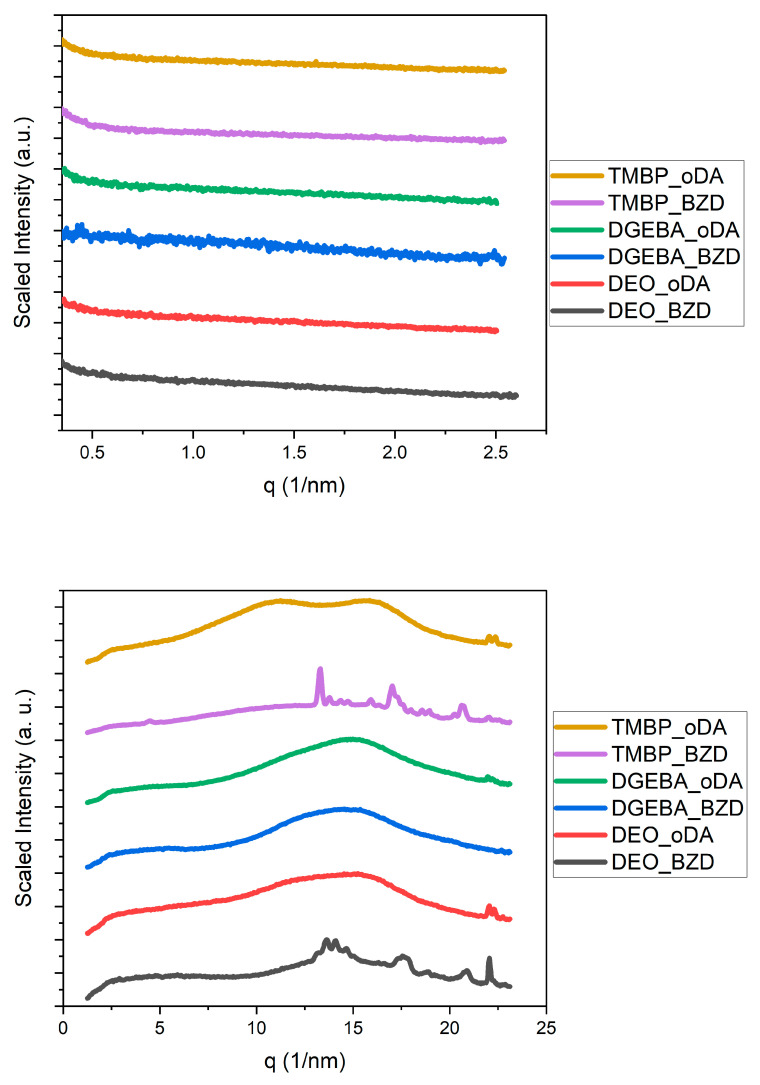
SAXS (**top**) and WAXS (**bottom**) patterns of TMBP, DGEBA, and DEO cured with oDA and BZD.

To investigate whether any resin–hardener combination exhibited partial crystallinity or mesophases, small-angle X-ray scattering (SAXS) and wide-angle X-ray scattering (WAXS) measurements were conducted. The SAXS and WAXS patterns of all the epoxy resins were compared with each other for all the hardeners, as shown in Figure 7, Figure 8 and Figure 9.

Small-angle X-ray scattering (SAXS) revealed scattering patterns characteristic of amorphous polymer networks for all resin/curing-agent combinations. No distinct Bragg peaks indicative of long-range or even partial crystallinity were observed. The only exception was a diffraction peak for the TME4/DDS at approximately 1.4 nm^−1^, which exhibited a position and width similar to those reported in a previous study [78]. This suggests that local ordering has occurred in this case, although it did not significantly affect the TC. In contrast, all SAXS patterns for the various resin and hardener combinations were featureless.

The wide-angle X-ray scattering (WAXS) results were largely similar across all investigated formulations, exhibiting a broad and substantial amorphous halo between 10 and 20 nm^−1^. This indicates that the resins are amorphous and lack long-range ordering. It is important to note that the WAXS patterns of DGEBA closely resemble those of the epoxy resins TMBP, LCE-DP, and TME4, which have been reported to exhibit liquid crystalline behavior [25,29,30,65,66,67,68,72,73,74,75,76,77,78,79,80,81,82,83,84,122,123]. This suggests that all resin–hardener combinations are amorphous under the conditions investigated. These findings are consistent with the measured thermal conductivity values. Significant crystalline phases typically enhance phonon transport and result in higher TC; however, the absence of crystallinity observed here aligns with the lack of notable thermal conductivity. There are two significant exceptions: DEO and TMBP cured with benzidine. These two resin–hardener combinations display more complex WAXS patterns characterized by sharper diffraction peaks. The reason for this phenomenon is unclear and warrants further investigation. Since the TC of these resin formulations falls within the same range as that of the other investigated epoxy resins, it suggests that ordered phases in epoxy resins have minimal impact on TC. It is possible that benzidine is poorly soluble in DEO and TMBP, potentially leaving behind small crystallites during the curing reaction. However, no melting peak was observed in the DSC, indicating that if this is the case, the portion of these crystallites must be small.

## 4. Discussion

### 4.1. Overview

This study tested two main hypotheses regarding the intrinsic thermal conductivity (TC) of epoxy resins:

- Partial or liquid-crystalline ordering in certain epoxy networks may significantly increase TC (potentially up to ~1 W/(m·K)), as reported previously for TME4, TMBP, and LCE-DP [25,29,30,65,66,67,68,72,73,74,75,76,77,78,79,80,81,82,83,84,122,123].
- Measurement discrepancies: Our own results along with previous studies [39,110,111,112] indicate that the use of different thermal conductivity measurement methods can lead to substantial variability in reported TC values for the same substance. This variability can potentially obscure modest structural enhancements in crosslinked epoxies. It is assumed that this variability reflects measurement-specific limitations that are not yet fully understood, possibly due to unknown systematic errors.

### 4.2. Interpretation of Key Findings

#### 4.2.1. No Evidence of Partial Crystallinity or Thermal Conductivities Around 1 W/(m·K)

None of the examined formulations, including TME4-based systems, exhibited TC values approaching 1 W/(m·K). This contrasts with prior reports suggesting significantly enhanced conductivity due to mesophase formation [77,78,79,80,81,82,83,84]. While TME4-based systems displayed a relatively sharp SAXS feature near 1.4 nm^−1^, indicating some long-range density variation, this did not result in higher thermal conductivity. SAXS and WAXS patterns showed amorphous characteristics for all samples, with the exception of anomalies in TMBP or DEO cured with benzidine, which did not lead to increased TC. These results align with the broader understanding that covalent crosslinking restricts large-scale chain ordering in polymers, confining the investigated thermosets to a ~0.2–0.3 W/(m·K) TC range under typical curing conditions. Indeed, multiple studies on polyethylene have noted that achieving partial crystallinity becomes exceedingly difficult once network formation begins [58,59,60,61,62,63]. This may help explain why the elevated TC values attributed to partially crystalline structures in epoxy thermosets could not be reproduced in this study. This finding also suggests that if partial crystallinity were contributing to higher thermal conductivity, it was either not present or not sufficient to elevate the measured TC beyond the typical ~0.3 W/(m·K) observed in most epoxy networks. While the degree of crosslinking is challenging to fully characterize, it is expected to limit the formation of ordered structures and the associated gains in conductivity.

#### 4.2.2. Method-Dependent Variations Overshadow Subtle Trends

Comparisons of LFA, MTPS, TDTR, and D-TOPS revealed that each technique can yield significantly different absolute TC values, often differing by 0.1–0.15 W/(m·K), which is comparable to the magnitude of the improvements attributed to liquid crystalline phases in the literature [25,29,30,62,63,64,65,69,70,71,72,73,113,114]. Consequently, variations in thermal conductivity measured by different methods often overshadow differences among formulations. This highlights the importance of independent confirmation, particularly when unusually high values are reported. Across literature reports for the same resin formulation, differences can be even greater; for example, a discrepancy of over 0.4 W/(m·K) has been reported for DGEBA cured with DDS [74,76,85,86,87,88,89,90,91,92,93,94,95,96,97,98,99,100,101,102,103,104,105,106,107,108,109]. This demonstrates that such comparisons can complicate the interpretation of measurement results. Given the magnitude of method-dependent variability observed here, it is advisable that structure–property relationships in epoxy resins focus on TC differences of at least 0.1 W/(m·K) and ideally be verified using multiple measurement approaches with varying sample preparation requirements.

Across the curing agents and schedules investigated in this study, no significant variation in thermal conductivity was observed within the measurement uncertainty. The available scattering data consistently indicated amorphous patterns. Based on these results and the narrow practical curing temperature window (dictated by melting points, volatility, reactivity, and degradation), we found no evidence that different curing temperatures would stabilize liquid crystalline order in these systems.

#### 4.2.3. Rigid vs. Flexible Structures

Although aromatic, rigid monomers (e.g., TMBP, LCE-DP) and aromatic curing agents (e.g., DDS, BZD, oDa) yielded slightly higher thermal conductivity (TC) than their flexible aliphatic counterparts (BDO, DDO), the effect remained modest. No formulation exceeded ~0.3 W/(m·K), suggesting that backbone rigidity alone is insufficient to achieve significant conductivity improvements without additional mechanisms, such as true crystallinity or high filler loading. This result may also align with the slight TC enhancements reported in several studies on LC epoxy resins [25,29,30,62,63,64,65,69,70,71,72,73,113,114].

### 4.3. Implications and Future Directions

Revisiting Literature Claims: Our findings indicate that reports of unusually high thermal conductivity (TC) values in liquid crystal (LC) epoxies should be approached with caution. We were unable to reproduce these values under controlled conditions in our study, and SAXS/WAXS measurements primarily suggested amorphous networks. Crosslinking constraints remain a critical factor; while the degree of crosslinking is challenging to fully characterize, the overall evidence suggests that LC order in epoxy thermosets is unlikely to lead to significant conductivity enhancements. Although POM images are frequently reported in this field, we believe that future experiments would benefit more from complementary scattering techniques that provide quantitative data.

Standardizing Measurement Protocols: Given the strong dependency of measured TC values on the measurement method, future research would benefit from standardized protocols or cross-verification across multiple techniques.

Toward Higher Intrinsic TC: Achieving substantial intrinsic conductivity improvements may necessitate entirely new approaches beyond ordered domains in crosslinked systems.

## Figures and Tables

**Table 1:** Curing conditions for DGEBA and TMBP epoxy resins with BDO, DDO and DDS.

Epoxy Monomer	Hardener	Mixing Temperature	Catalyst	# of Samples	Curing in Air
3.8 g (10 mmol; 1 eq.) DGEBA	0.9 g (10 mmol; 1 eq.) 1,4-Butanediol	130 °C	19 µL 1-MI	1	6 h/150 °C 24 h 180 °C
3.8 g (10 mmol; 1 eq.) DGEBA	1.74 g (10 mmol; 1 eq.) 1,4-Decanediol	130 °C	22,5 µL 1-MI	1	6 h/150 °C 24 h 180 °C
6.1 g (16 mmol; 1 eq.) DGEBA	4.0 g (16 mmol; 1 eq.) DDS	170 °C	-	2	2 h 180 °C
20.0 g (56 mmol; 1 eq.) TMBP	5.0 g (55 mmol; 1 eq.) 1,4-Butanediol	130 °C	75 µL 1-MI	3	2 h 150 °C 20 h 180 °C 24 h 200 °C
17.0 g (48 mmol; 1 eq.) TMBP	8.0 g (46 mmol; 1 eq.) 1,10-Decanediol	130 °C	75 µL 1-MI	3	2 h 150 °C 20 h 180 °C 24 h 200 °C
9.0 g (25 mmol; 1 eq.) TMBP	6.0 g (24 mmol; 1 eq.) DDS	170 °C	-	2	8 h 180 °C

**Table 2:** Thermal diffusivity (TD) values measured via LFA and D-TOPS, as well as the thermal effusivity (TE) values measured via MTPS and TDTR, including the corresponding heat capacities (C_p_), densities (d), and glass transition temperatures (T_g_) for each sample.

Sample	$\mathrm{TE}\;\mathrm{MTPS}\;\left[\frac{kWs^{1/2}}{m^{2}K}\right]$	$\mathrm{TE}\;\mathrm{TDTR}\;\left[\frac{kWs^{1/2}}{m^{2}K}\right]$	TD LFA [mm^2^/s]	TD D-TOPS [mm^2^/s]	C_p_ DSC [J/gK]	ρ [g/mL]	T_g_ [°C]
DGEBA/BDO	0.74	0.77	0.10	-	2.15	1.18	13
DGEBA/DDO	0.70	-	0.10	-	2.28	1.14	5
DGEBA/DDS	0.69	0.48	0.13	0.13	1.42	1.28	138
DGEBA/BZD	-	0.51	-	0.12	1.33	1.28	180
DGEBA/oDA	-	0.52	0.17	0.13	1.12	1.23	102
DEO/DDS	-	0.52	-	0.14	1.32	1.30	68
DEO/BZD	-	0.61	-	0.16	1.62	1.20	39
DEO/oDA	-	0.65	-	0.14	1.57	1.27	57
TMBP/BDO	0.73	-	0.13	-	1.78	1.17	25
TMBP/DDO	0.75	-	0.12	-	1.68	1.12	19
TMBP/DDS	0.71	0.57	0.15	0.14	1.12	1.25	148
TMBP/BZD	-	0.59	0.22	0.15	1.27	1.26	89
TMBP/oDA	-	0.65	0.18	0.16	1.14	1.23	120
LCE-DP/BDO	0.72	0.68	0.17	0.13	1.56	1.26	44
LCE-DP/DDO	0.74	0.67	0.16	0.13	1.62	1.20	48
LCE-DP/DDS	-	0.58	0.20	0.20	1.13	1.31	145
TME4/DDS	0.69	0.63	0.18	0.19	1.17	1.33	123

## Data Availability

The original contributions presented in the study are included in the article, further inquiries can be directed to the corresponding author.
